# Surgeon Performed Ultrasound for Diagnosis of Intussusception - A Pilot Study

**DOI:** 10.24908/pocus.v6i1.14760

**Published:** 2021-04-22

**Authors:** Soundappan S V Soundappan, Albert Lam, Lawrence Lam, Danny Cass, Andrew J A Holland, Jonathan Karpelowsky

**Affiliations:** 1 Department of Surgery, The Children's Hospital at Westmead, Sydney medical School, University of Sydney Sydney Australia; 2 Department of Medical Imaging, The Children’s Hospital at Westmead, Sydney, Sydney Medical school, University of Sydney Sydney Australia; 3 Vice President (Academic), Tung Wah College Hong Kong

**Keywords:** Intussusception, Point of care ultrasound, surgeon performed ultrasound, diagnosis

## Abstract

**Aim: **To study the diagnostic accuracy of surgeon performed ultrasound (SPU) in the diagnosis of children presenting with clinical suspicion of intussusception to a tertiary paediatric facility in NSW, Australia. **Methods: **Children under the age of 16 presenting to the emergency department with clinical features suggestive of intussusception were recruited. After obtaining consent SPU was performed by a Paediatric surgeon. All patients subsequently had an ultrasound performed in radiology department (RPU) on which management was based. Diagnosis and images of SPU were reviewed by an independent radiologist blinded to results of the formal study. **Results: **Of 7 children enrolled 5 were male. Age ranged from 3 months to 7 years (mean 2.64, SD 2.282), weight from 5.2kgs to 25.2kgs (mean 13.69, SD 6.721). Five out of the 7 children presented during day hours i.e. 8a.m.-5 p.m. (mean 12.72, SD 4.049). Mean time to SPU was 6.3 hours (SD7.1) and RPU was 8.3 hours (SD 7.6). SPU was earlier by 2 hours and correlation between SPU and RPU was 100 percent. **Conclusion: **SPU for intussusception can be performed early and accurately. Surgeons should train and use ultrasound as a reliable tool in evaluating the child with suspected intussusception.

## Introduction

Intussusception is an abdominal emergency that affects all ages but infants most commonly and can cause significant morbidity and mortality if missed 1, 2. Clinical presentation of intussusception can be varied and non-specific and imaging (primarily ultrasound) is usually performed to confirm diagnosis 1. Abdominal radiographs may be non-specific and contrast studies involve ionising radiation. Ultrasound (US) has evolved as the gold standard imaging for diagnosis because of its high accuracy 2. It is also used to guide reduction of intussusception in some centres while in most institutions air enema under fluoroscopy is still the treatment of choice 3, 4, 5. Surgery is reserved for failed pneumatic reductions, delayed presentations or complications such as perforation. We have anecdotally noticed many infants with intussusception present after hours, as have other authors 1, when sonographers may not be available and have to be recalled. This may contribute to delay in diagnosis, treatment and also has financial implications for the institution. A surgeon performed point of care ultrasound (POCUS) at the time of clinical examination should lead to earlier diagnosis and treatment. This pilot study sought to assess diagnostic accuracy and timeliness of SPU compared to Radiology Performed Ultrasound (RPU) in a tertiary paediatric hospital in NSW, Australia.

## Methods

This was an ethics approved (HREC 11/CHW/27) study performed at The Children’s hospital at Westmead, a tertiary Paediatric hospital in New South Wales Australia. The hospital serves a population of 2.02 million (0-16) with 50,000 presentations to the emergency annually. Infants with a suspected diagnosis of intussusception referred to the surgical department were considered for the study. The surgical registrar offered the study to the families after assessing the infant. Ultrasound request for RPU was placed after surgical assessment and surgeon was also contacted at same time for SPU.US were performed by only one surgeon (first author) if the family agreed to participate in the study. RPU was performed by the sonographer who was on for the day. The first author has been performing FAST scans for 17 years and ultrasound guided central venous cannulation for 10 years. All enrolled children also had a RPU on which treatment was based. Study population was a convenience sample as we depended on the assessing doctor to contact the surgeon and it is likely the surgeon was not contacted for all eligible cases.

**Figure 1:**
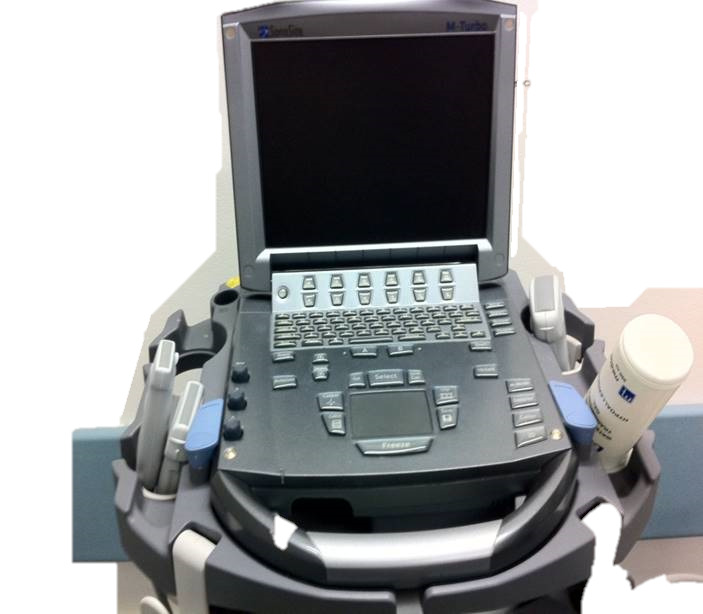
Sonosite Turbo machine used for the study

Technique: Sonosite M-Turbo mobile ultrasound (Sonosite Inc ,Washington, USA) was used for the studies. Three different probes were used as needed- C60 2-5MHz curved probe with 30cm scan depth for older children (>6years), C11 5-8MHz curved probe with scan depth of 10cm for children under 6 and HFL38 6-13MHz linear transducer with scan depth 6cm (Figure 1). With the patient in supine position, curvilinear transducer was used to examine the whole abdomen looking for any masses of non-peristaltic bowel. If a mass suggestive of an intussusception was identified, a linear probe was used to demonstrate a target sign in the transverse axis and/or a pseudokidney sign in long axis and its dimensions measured. Presence or absence of fluid between bowel loops was documented. Doppler study was performed to document vascularity within the mass. RPU was performed with ultrasound equipment in radiology department not the Sonosite used by the surgeon. 

A radiologist blinded to the results of RPU reviewed all SPU studies to assess adequacy of films and diagnosis. Gold standard was the result of RPU. Patient details and US findings were recorded on a predesigned data collection form and entered into an Excel file (Microsoft, Redmond, WA). Time to scan from referral time was documented. Patient outcomes were also recorded, including admission for observation, operative intervention or discharged (online Table S1). Readmissions and other investigations performed were recorded. Data was analysed using the IBM SPSS V22.0 statistical software. Descriptive analyses were applied to the data using means, standard deviations, median, range, frequencies, and percentages in accordance to the nature of the variables. Comparisons of means were conducted using paired t-tests for continuous variables. 

## Results

Over a one year period 7 children with suspected intussusception were recruited into the study. Five were boys. Age ranged from 3 months to 7 years (mean 2.64, SD 2.282), weight from 5.2kgs to 25.2kgs (mean 13.69, SD 6.721). Five out of the 7 children presented during day hours (mean 12.72, SD 4.049). Mean time of presentation of infant was midday. Mean hour of the day for SPU was 12.3 hours (SD 2.9) and RPU was 14.3 hours (SD 2.7).Mean time to SPU was 6.29 hours and RPU was 8.28 hours from time of presentation to ED. One child had a second presentation within a week of the initial presentation. Mean time difference was 2 hours in favour of SPU. Paired t-test comparing time of surgeon performed ultrasound and radiology performed ultrasound was significantly p=0.003 in favour of SPU.

**Figure 2:**
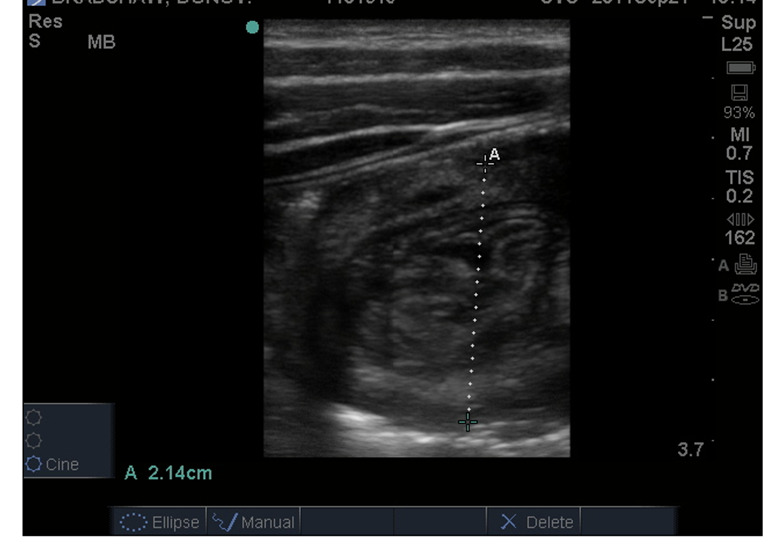
Intussusception diagnosed on SPU.

Three out of the seven cases had intussusception (Figure 2) correctly diagnosed by both surgeon and radiology, with no false positives or false negatives. All three cases had target and pseudokidney sign demonstrated and were successfully reduced by air enema. The child with two presentations had both successful air enemas on both occasions. An independent radiologist agreed with the diagnosis of SPU in all cases (Kappa 1.00, p=0.008).

## Discussion

Intussusception is the commonest surgical emergency in infancy 5. US has emerged as the investigation of choice for diagnosis and has a sensitivity of 98-100% and specificity of 88-100% 1, 6, 7. We compared SPU to RPU in this study and demonstrated SPU could be performed earlier and was accurate in diagnosis when performed by an experienced consultant surgeon. False positive studies are uncommon and usually due to faecal matter mistaken for intussusception 7. Only one of the previous studies on POCUS has been by Paediatric surgeons^8^ and our study adds to the existing literature supporting POCUS for diagnosis of intussusception.

Over the last decade point of care ultrasound (POCUS) has become routine in many paediatric emergencies for a variety of clinical scenarios 6. Paediatric surgeons however, have been slow to adopt POCUS and this study looked at use of SPU to diagnose intussusception. Inspite of the low numbers the surgeon’s interpretation of the studies matched the radiology diagnosis in all the cases. POCUS by clinician for diagnosis of intussusception has been shown to reduce time in making diagnosis aiding higher success of reduction 6, 7. In our study SPU was performed 2 hours earlier than RPU. The time difference could have been longer if SPU was performed at the time of assessment by the surgical registrar. It was performed by a consultant amidst the rest of his clinical duties SPU is a natural extension of clinical examination and may decrease the need to call in a sonographer for diagnosis. Brian et al. retrospectively reviewed POCUS diagnosed intussusceptions over a five year period and demonstrated small bowel intussusceptions were more common than classical ileocolic intussusception 9. In our study all cases were ileocolic intussusceptions. A recent meta-analysis analysed 30 studies on diagnosis of intussusception at all ages of which 7 were POCUS based studies. They concluded the diagnostic accuracy of POCUS was not significantly different from that of RPU 10. 

Limitations of our study include the small numbers and scan being performed by a surgeon who performed them along with other clinical duties. This may have caused some delay in SPU. Diagnosis by SPU may avoid calling in a sonographer but a radiographer and radiologist would still need to be called in for the pneumatic reduction. An extension of the use of SPU would be ultrasound guided reduction that would obviate the need to call in radiology staff and reduce radiation exposure as demonstrated by S. Gfroerer and colleagues 8. POCUS has demonstrated increased patient satisfaction as there is more interaction with the clinician, reduced patient movement and earlier diagnosis 7, 11.

This study is the first of its kind in Australasia demonstrating use of ultrasound by a surgeon in diagnosis of intussusception and we hope will stimulate use in emergency departments by emergency physicians and surgeons.

## Disclosures

The authors have no conflicts of interest to declare.

## Supplementary Material

Table S1 Comparing SPU and RPU for intussusception. Time to SPU or PRU is hours since presentation to emergency department
