# Supplementary material for: Surgeon Performed Ultrasound for Diagnosis of Intussusception - A Pilot Study
Source: POCUS J. 2021 Apr 22;6(1):33–5. doi: 10.24908/pocus.v6i1.14760 (PMC9979933; doi:10.24908/pocus.v6i1.14760)
Supplement: Table S1 [file pocusj-06-14760-s001.pdf]

Table S1. Comparing SPU and RPU for intussusception. Time to SPU or PRU is hours since presentation to emergency department

S.No	Age	Time to SPU (hours)	Diagnosis (Y/N)	Vascularity in mass (Y/N)	Time to RPU (hours)	Diagnosis (Y/N)	Vascularity (Y/N)	Time difference between SPU and RPU (hours)
1	3 months	4.02	n		5.21			1.19
2	3yrs	1.53	n		1.88	N		0.35
3	2 months	3.25	N		6.04	N		2.79
4	7	0.72	y	y	1.83	y	y	1.19
5	2	1.69	y	y	4.83	y	y	3.14
6	3	14.48	y	y	16.75	y	y	2.29
7	3	18.38	n		21.38	n		3
